# Supplementary material for: Resolving the full spectrum of human genome variation using Linked-Reads
Source: Genome Res. 2019 Apr;29(4):635–45. doi: 10.1101/gr.234443.118 (PMC6442396; doi:10.1101/gr.234443.118)
Supplement: Supplemental Material [file supp_gr.234443.118_Supplemental_longranger-2.2.zip › longranger-2.2.tar.bz2!/lr2.2-public/lib/stan/cmdstan/stan/doc/api/html/structstan_1_1math_1_1array__builder.html]

Stan: stan::math::array\_builder< T > Struct Template Reference


|  |  |
| --- | --- |
|  | Stan  2.5.0  probability, sampling & optimization |


- Main Page
- Related Pages
- Namespaces
- Classes
- Files

- Class List
- Class Index
- Class Hierarchy
- Class Members

All Classes Namespaces Files Functions Variables Typedefs Enumerations Enumerator Friends Macros Pages

- stan
- math
- array\_builder

Public Member Functions |
Public Attributes |
List of all members

stan::math::array\_builder< T > Struct Template Reference

Structure for building up arrays in an expression (rather than in statements) using an argumentchaining add() method and a getter method array() to return the result.
More...

`#include <array_builder.hpp>`

|  |  |
| --- | --- |
| Public Member Functions | |
|  | array\_builder () |
|  | |
| template<typename F > | |
| array\_builder & | add (const F &u) |
|  | |
| std::vector< T > | array () |
|  | |

|  |  |
| --- | --- |
| Public Attributes | |
| std::vector< T > | x\_ |
|  | |

## Detailed Description

### template<typename T> struct stan::math::array\_builder< T >

Structure for building up arrays in an expression (rather than in statements) using an argumentchaining add() method and a getter method array() to return the result.

Definition at line 16 of file array\_builder.hpp.

## Constructor & Destructor Documentation

template<typename T >

|  |  |  |  |  |
| --- | --- | --- | --- | --- |
| stan::math::array\_builder< T >::array\_builder | ( |  | ) |  |

inline

Definition at line 18 of file array\_builder.hpp.

## Member Function Documentation

template<typename T >

template<typename F >

|  |  |  |  |  |  |
| --- | --- | --- | --- | --- | --- |
| array\_builder& stan::math::array\_builder< T >::add | ( | const F & | *u* | ) |  |

inline

Definition at line 20 of file array\_builder.hpp.

template<typename T >

|  |  |  |  |  |
| --- | --- | --- | --- | --- |
| std::vector<T> stan::math::array\_builder< T >::array | ( |  | ) |  |

inline

Definition at line 26 of file array\_builder.hpp.

## Member Data Documentation

template<typename T >

std::vector<T> stan::math::array\_builder< T >::x\_

Definition at line 17 of file array\_builder.hpp.

---

The documentation for this struct was generated from the following file:

- src/stan/math/matrix/array\_builder.hpp


---

[ Stan Home Page ]

*© 2011–2014,
Stan Development Team.*
